# Health-related quality of life varies in different respiratory disorders: a multi-case control population based study

**DOI:** 10.1186/s12890-019-0796-8

**Published:** 2019-02-07

**Authors:** Veronica Cappa, Alessandro Marcon, Gianfranco Di Gennaro, Liliya Chamitava, Lucia Cazzoletti, Cristina Bombieri, Morena Nicolis, Luigi Perbellini, Silvia Sembeni, Roberto de Marco, Francesco Spelta, Marcello Ferrari, Maria Elisabetta Zanolin

**Affiliations:** 1 Unit of Epidemiology and Medical Statistics, Department of Diagnostics and Public Health, University of Verona, c/o Istituti Biologici II, Strada Le Grazie 8, 37134 Verona, Italy; 2 Unit of Hygiene and Preventive, Environmental and Occupational Medicine, Department of Diagnostics and Public Health, University of Verona, Verona, Italy; 3 Department of Pathology and Diagnostics, University Hospital of Verona, Verona, Italy; 4 Unit of Biology and Genetics, Department of Neuroscience, Biomedicine and Movement, University of Verona, Verona, Italy; 5 Unit of Occupational Medicine, Azienda Ospedaliero Universitaria di Verona, Verona, Italy; 6 Department of Medicine, Unit of Respiratory Medicine, University of Verona, Verona, Italy

**Keywords:** Quality of life, Allergy, Asthma, COPD, Clinical epidemiology

## Abstract

**Background and objective:**

Health-related quality of life (HRQL) in respiratory diseases has been generally investigated in clinical settings, focusing on a single disorder. In this study on a general population sample, we assessed the relationship between HRQL and several respiratory diseases studied simultaneously (COPD, current (CA) and past (PA) asthma, allergic (AR) and non-allergic (NAR) rhinitis and chronic bronchitis (CB).

**Methods:**

Controls (*n* = 328) and cases of NAR (*n* = 95), AR (*n* = 163), CB (*n* = 48), CA (*n* = 224), PA (*n* = 126) and COPD (*n* = 28) were recruited in the centre of Verona in the frame of the Italian multi-case control GEIRD (Gene Environment Interactions in Respiratory Diseases) study; HRQL was measured through the SF-36 questionnaire. The relationships between HRQL (in terms of Physical (PCS) and Mental Component Scores (MCS)), respiratory diseases, and covariates were evaluated.

**Results:**

With respect to controls, the adjusted PCS median score was worse in subjects suffering from current asthma (− 1.7; 95%CI:-2.8;-0.6), CB (− 3.8; 95%CI:-5.7;-1.9), and COPD (− 5.6; 95%CI:-8.1;-3.1). MCS was worse in current asthmatics (− 2.2; 95%CI:-4.1;-0.3), CB (− 5.5; 95%CI:-8.7;-2.2), and COPD cases (− 4.6; 95%CI:-8.8;-0.5) as well.

**Conclusions:**

To our knowledge, this is the first study in the general population that analyzed HRQL performing a simultaneous comparison of HRLQ in several respiratory disorders. We found that subjects suffering from COPD, CA, and CB had the poorest HRQL. Clinicians should carefully consider the possible impact of respiratory disorders as CB and not only that of CA and COPD.

**Electronic supplementary material:**

The online version of this article (10.1186/s12890-019-0796-8) contains supplementary material, which is available to authorized users.

## Summary at a glance

We conducted a population-based study investigating the differences in HRQL across the most common chronic respiratory disorders. Unlike previous studies, we were able to consider several diseases simultaneously by using a multicase-control design. COPD, asthma and chronic bronchitis showed worse physical and mental SF-36 scores than controls.

## Introduction

In chronic illnesses, Health-related Quality of Life (HRQL, which daily activities people can perform and how they feel) is a primary concern and is often used as the outcome in both clinical trials and observational studies [1]. In addition, as people generally seek health care only when they feel unhealthy, self-perception of health is a predictive of the future burden on the health care delivery system [2, 3].

Asthma is a critical public health problem worldwide, affecting people of all ages and both sexes [4, 5]. It impairs school and work performance [6] as well as physical and mental quality of life [3, 4, 7].

Chronic obstructive pulmonary disease (COPD) is characterized by persistent airflow limitation, associated with an enhanced chronic inflammatory response in the airways and the lungs to noxious particles or gases. Frequently, patients suffering from COPD have impaired daily activities, often in association with symptoms of dyspnea and fatigue [8, 9]. Also subjects with chronic bronchitis (CB) may present poor mental health [10–12]. Rhinitis is an inflammation of the lining of the nose and is characterized by nasal symptoms, including rhinorrhea, sneezing, nasal blockage and/or itching of the nose [13]. Allergic rhinitis (AR), a symptomatic disorder of the nose induced after allergen exposure, may be associated with daily activities reduction [14]. In addition, having an allergic reaction could cause significant fatigue and mood changes [15], impairment of cognitive functions [16, 17] depression, and anxiety [18, 19]. Although HRQL has been frequently assessed in subjects with AR [15, 19], there is a lack of epidemiological studies on non-allergic rhinitis (NAR) [20], especially on the quality of life of people suffering from it.

Subjects suffering from AR commonly have current asthma (CA) and the effect of AR alone cannot be disentangled. Therefore, it can be of interest to analyse the role of AR in subjects without asthma.

HRQL in respiratory diseases has been mainly investigated in a clinical setting, where usually subjects have a worse condition with respect to general population and, as a consequence, present a HQRL that does not correspond to real life [17, 20–22]. Moreover, most of previous studies focused on a single disorder, losing the chance to analyze the relative weight of the different respiratory disorders.

In the frame of the Gene Environment Interactions in Respiratory Diseases (GEIRD) survey, a multicase-control study in the general population, the aim of this paper was to assess the relationship between HRQL and COPD, asthma, CB, and rhinitis considered simultaneously.

## Methods

**Fig. 1:**
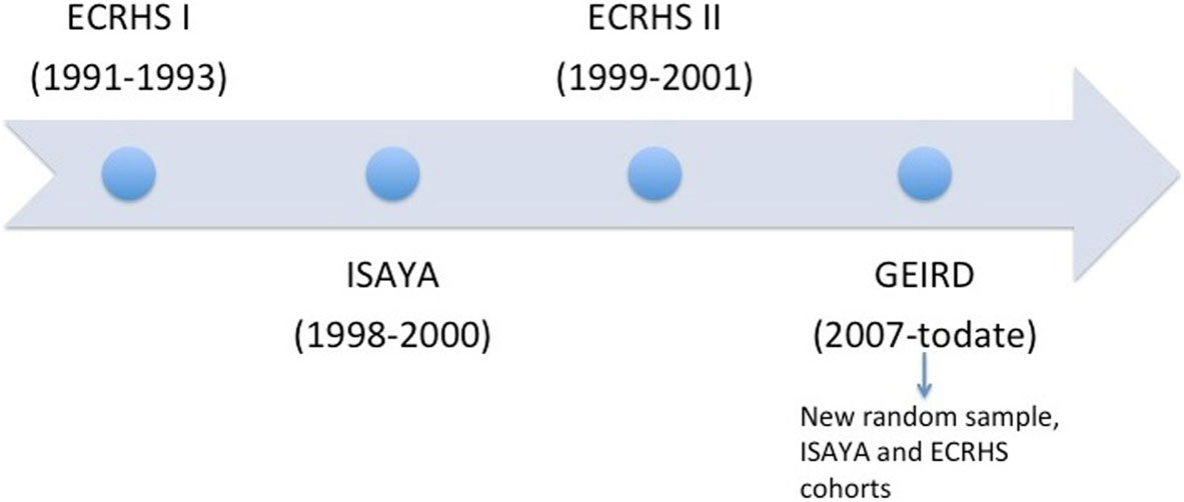
Timeline for the new random sample and the cohorts of the GEIRD study

The GEIRD project is an ongoing multicase-control study, coordinated by the Verona centre, involving seven Italian centres [23]. In the first stage of the study, new random samples and pre-existing cohorts (the Italian Study on Asthma in Young Adults (ISAYA) [24] and the Italian arm of the European Community Respiratory Health Survey (ECRHS) [25]) from the general population (20–64 years, male/female = 1/1) were mailed a screening questionnaire on respiratory symptoms (Fig. 1). In particular, for the new random sample, 3000 subjects aged 20–44 and 1000 subjects aged 45–64, male/females =1/1, from the general population were selected. In the second stage of GEIRD, on the basis of answers to the screening questionnaire (Additional file 1 (a)), all probable cases of asthma and COPD/ CB, a sample of probable cases of AR (44%), other condition (39%), and controls (61%), were invited to clinics to be phenotyped [26]. During the clinical visit, each subject underwent a computer-assisted clinical interview, lung function [27, 28] and allergological tests [29] (Additional file 1 (b)), and the 36-item Short Form (SF-36) questionnaire. All the protocols were in agreement with the international guidelines and can be found on the GEIRD website (www.geird.org) [30]. Ethical approval was obtained in each centre from its appropriate ethics committee, and written consent was obtained from each participant.

In this paper, only the data of the Verona centre were considered, because the data collection was at an initial stage in the other centres.

### Quality of life questionnaire – The SF-36

As a part of the GEIRD core protocol, all the participants’ HRQL was assessed using SF-36 questionnaire version 1.6, which is a generic quality of life, self-administered measure containing 36 items [27, 31]. Physical Component Summary (PCS) and Mental Component Summary (MCS) measures were calculated. Missing data were treated as recommended in the SF-36 manual and interpretation guide [28].

### Identification of cases and controls in clinics

The subjects were hierarchically classified as follows:

- 28 cases of COPD (he/she had post-bronchodilator FEV1/FVC < LLN or < 70%);
- 224 cases of current asthma (“CA”, he/she reported lifetime asthma; or he/she reported asthma-like symptoms/medicines in the last 12 months and had (1): a positive methacholine challenge test with PD20 < 1 mg or (2) pre-bronchodilator FEV1/FVC < 70% or < LLN with a positive reversibility test (i.e. FEV1 > 12% and > 200 mL after the administration of 400 μg of salbutamol); all cases already defined as COPD cases could not be defined as CA cases;
- 126 cases of past asthma (“PA”, he/she reported lifetime asthma but did not fulfil the criteria for CA); all cases already defined as COPD cases could not be defined as PA cases;
- 48 cases of CB (he/she was not a COPD or CA/PA case and he/she reported chronic cough or phlegm (> 3 months/year for at least 2 years));
- 163 cases of allergic rhinitis (AR) and 95 non-allergic rhinitis (“NAR”) (he/she was not a COPD, CA/PA, BC case and he/she had nasal allergies or nasal problems in the presence of animal(s), pollens, dust plus negative (NAR) or positive (AR) skin prick test);
- 328 controls (subjects without any nasal/respiratory symptoms/conditions reported in the clinical questionnaire neither in the clinic nor in the screening questionnaire).

The Additional file 2 shows the details of this hierarchical classification and the overlapping among cases.

### Potential determinants of HRQL and covariates

The relationship between case-control status (COPD, CA, PA, AR, NAR, CB and controls, the independent variable) and HRQL (the dependent variable) was investigated. We took into account the following potential confounders: gender, age (years), body mass index (BMI, kg/m2), education level (low if a subject had completed full-time education before the age of 16, high otherwise), smoking habits (never smoker, past smoker = ever smoker who did not smoke in the last month, current smoker = subject who smoked in the last month), presence/absence of at least one non-respiratory comorbidity (gastritis, stomach ulcer, gastroesophageal reflux, hiatal hernia, esophagitis, osteoporosis, gout, arthritis, osteoarthritis, pulmonary embolism, diabetes, stroke, cancer), presence/absence of at least one heart disease (heart attack (coronary thrombosis), angina, arrhythmia, hypertension and other heart problems).

The analyses were also adjusted for study sample/cohort (ECRHS Italy, ISAYA, new random sample) and calendar period, which divided the study period into semesters, starting from the beginning of stage 2 (April 2008). The latter covariate took into account both seasonality (April to September, the “warm/hot” season in Italy, compared to October to March, the “cool/cold” season) and potential temporal trends.

### Statistical analysis

Data were summarized as counts and percentages, means (standard deviation (SD)) and medians (interquartile difference (IQD)). The Chi-square and the Kruskal-Wallis tests were used to investigate differences in variables among respiratory conditions, where appropriate. As scores were not normally distributed, differences in the crude medians of PCS and MCS across cases and controls were tested using the Kruskal-Wallis test.

Quantile regression models [32] were applied to study the relationship between HRQL and respiratory diseases, controlling for the other potential determinants, estimating conditional medians of the response variables (PCS and MCS). The quantile regression coefficients are interpreted as the ordinary regression ones and they indicate the change in the dependent variable for every one unit change (or category for nominal variables) in each covariate [33]. As higher PCS and MCS scores are index of better HRQL, a negative regression coefficient indicates that an increase in the independent variable worsens HRQL and vice versa.

Statistical analyses were performed using Stata Statistical Software: Release 14.0 (www.stata.com).

## Results

### Sample characteristics

Out of 1046 subjects who went to the Verona clinic for tests, 1012 (96.7%) correctly filled-in SF-36 questionnaire and 34 (3.3%) did not (6 did not fill it in completely, 28 did not answer more than three questions). When compared to subjects with an available quality of life questionnaire, subjects without SF-36 were similar to the others, except for a lower education level (41.4% vs 20.4%, *p* = 0.004), and age (47.1 vs. 44.0, *p* = 0.047) (Table 1).

**Table 1:** Characteristics of subjects who did and did not complete the SF-36

	SF-36 available (*n* = 1012)	SF-36 not available (*n* = 34)	*p*-value
Gender (female), n (%)	520 (51.4%)	21 (61.8%)	0.233
Age years, mean (sd)	43.96 (9.8)	47.11 (10.4)	0.047
BMI kg/m2, mean (sd)	24.8 (4.3)	24.5(4.2)	0.488
Education (low), n (%)	209 (20.7%)	14 (41.2%)	0.004
Smoking habits, n(%)			0.094
never smokers	500 (49.5%)	12 (35.3%)	
past smokers	277 (27.4%)	15 (44.1%)	
current smokers	233 (23.7%)	8 (22.2%)	
Non-resp. comorbidities, n (%)	336 (33.3%)	9 (26.5%)	0.403
Cardiac comorbidities, n (%)	148 (14.7%)	5 (14.7%)	0.997
Phenotypes^a^, n (%)			0.448
controls	328 (32.4%)	15 (44.1%)	
NAR	95 (9.4%)	6 (17.6%)	
AR	163 (16.1%)	5 (14.7%)	
CB	48 (4.7%)	1 (2.9%)	
PA	126 (12.4%)	1 (2.9%)	
CA	224 (22.1%)	5 (14.7%)	
COPD	28 (2.8%)	1 (2.9%)	

^a^*NAR* non-allergic rhinitis, *AR* allergic rhinitis, *CB* chronic bronchitis, *PA* past asthma, *CA* current asthma, *COPD* chronic obstructive pulmonary disease

Out of 1012 subjects who filled-in the SF-36 questionnaire correctly, 328 (32.4%) were controls, 95 (9.4%) and 163 (16.1%) were NAR and AR respectively, 48 (4.7%) suffered from CB, 126 (12.4%) and 224 (22.1%) were PA and CA cases respectively, and 28 (2.8%) were COPD cases (comorbidities among respiratory diseases are reported in Additional file 2: Figures S1-S4).

The characteristics of the 1012 subjects included in the main analyses are described in Table 2. Cases of NAR and PA were prevalently females (61.1 and 57.9% respectively), while cases of COPD were prevalently males (75%; *p* = 0.021). With respect to the other cases and controls, subjects suffering from COPD were older (52.2 years), had a lower educational status (35.7%) and presented more often other non-respiratory comorbidities (50%). Controls, AR and past asthmatics were mainly never smokers (52.8, 58.9 and 53.2% respectively), while CB and COPD cases had the highest percentage of current smokers (37.5 and 32.1%). Cardiac comorbidities were reported in higher percentages in NAR (25.2%) and CB (22.9%) subjects than other cases and controls. Non-respiratory comorbidities were more frequent in COPD and CB subjects (50.0 and 41.7% respectively).

**Table 2:** Characteristics of cases^a^ and controls who filled-in SF-36

	Controls	NAR	AR	CB	PA	CA	COPD	*p*-value^b^
n (%)	328 (32.4%)	95 (9.5%)	163 (16.1%)	48 (4.7%)	126 (12.4%)	224 (22.1%)	28 (2.8%)	
Gender (female), n (%)	168 (51.2)	58 (61.1)	79 (48.5)	26 (54.2)	73 (57.9)	109 (48.7)	7 (25.0)	0.021
Age years, mean (sd)	45.3 (9.6)	44.8 (9.7)	42.9 (10.7)	43.8 (9.6)	43.1 (9.4)	41.9 (9.4)	52.2 (8.5)	< 0.001
BMI kg/m2, mean (sd)	25.0 (4.3)	24.3 (4.4)	24.3 (4.0)	24.8 (4.3)	24.9 (4.7)	24.8 (4.2)	25.8 (3.4)	0.231
Education (low), n (%)	78 (23.8)	24 (25.3)	25 (15.3)	18 (37.5)	15 (12.0)	39 (17.4)	10 (35.7)	< 0.001
Smoking habits, n(%)								< 0.001
never smokers	172 (52.8)	34 (35.8)	96 (58.9)	22 (45.8)	67 (53.2)	99 (44.2)	10 (35.7)	
past smokers	98 (30.1)	33 (34.7)	38 (23.3)	8 (16.7)	31 (24.6)	60 (26.8)	9 (32.1)	
current smokers	56 (17.2)	28 (29.8)	29 (17.8)	18 (37.5)	28 (22.2)	65 (29.0)	9 (32.1)	
Non-resp. comorbidities, n (%)	74 (22.6)	45 (47.9)	55 (33.7)	20 (41.7)	47 (37.6)	81 (36.3)	14 (50.0)	< 0.001
Cardiac comorbidities, n (%)	33 (10.2)	24 (25.2)	31 (19.0)	11 (22.9)	17 (13.6)	28 (12.5)	4 (14.3)	0.010
FEV1% predicted, mean (sd)	101.9(11.3)	100.9(12.6)	100.3 (12.0)	96.8(10.7)	98.9(12.4)	97.0 (12.3)	78.6 (15.5)	< 0.001

^a^*NAR* non-allergic rhinitis, *AR* allergic rhinitis, *CB* other respiratory condition, *PA* past asthma, *CA* current asthma, *COPD* chronic obstructive pulmonary disease

^b^Differences in categorical variables among phenotypes were tested by Pearson Chi-square test; differences in continuous variables among phenotypes were tested using Kruskal-Wallis test

### PCS and MCS scores

**Fig. 2:**
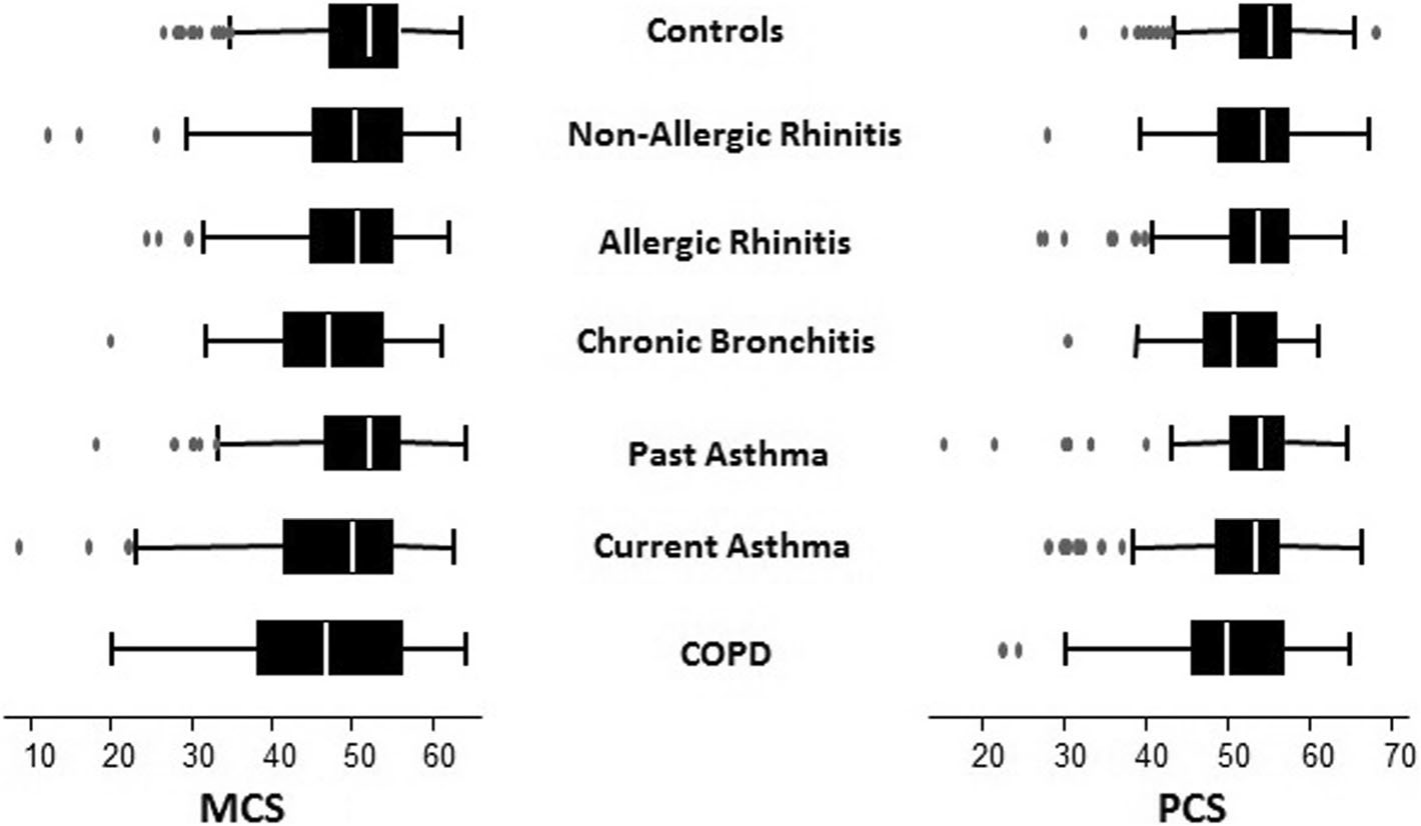
Physical and Mental SF-36 median scores and inter-quartile range by respiratory diseases

The crude median PCS score of controls (55.1, IQD: 51.8–57.7) was higher than those of subjects suffering from respiratory diseases (*p* < 0.001). In particular, the physical score of NAR, AR and PA cases was respectively 54.4, 53.9 and 54.2, while the scores of CA, CB and COPD cases were the lowest of the sample (53.6, IQD: 48.8–56.3; 51.0, IQD: 47.4–55-8; 48.9, IQD: 45.9–56.8 respectively) (Fig. 2).

Controls and cases of PA had the highest crude median MCS score (52.3, IQD: 47.6–56.1; 52.4, IQD: 46.9–55.9 respectively) followed by NAR, AR and CA (NAR 50.4, IQD: 45.2–56.4; AR 50.7, IQD: 45.1–55.0; CA 50.3, IQD: 41.9–54.9) (Fig. 2). Subjects who suffered from CB and COPD had the lowest MCS (CB 47.1, IQD: 41.7–53.8; COPD 46.8, IQD 38.6–56.3 respectively). Median MCS differed among controls and cases (*p* < 0.001).

The above-mentioned findings were confirmed when the outcomes (PCS and MCS) were adjusted for possible determinants and confounders (Table 3). In general, all the subjects suffering from respiratory diseases showed lower PCS scores than the controls. In particular, in subjects who suffered from CB the median score was 3.8, in current asthmatics 1.7 and COPD cases 5.6 statistically significantly lower than in controls (Table 3). Both AR and NAR did not show a significant difference in HRQL with respect to controls.

**Table 3:** Relationships^a^ between HRQL (in terms of PCS and MCS), respiratory diseases

	PCS	MCS
	Coeff. (95%CI)	Coeff. (95%CI)
Respiratory diseases^b^ (vs *controls*)		
NAR	0.1 (−1.4;1.5)	−0.8 (−3.3;1.7)
AR	−0.8 (−2.0;0.4)	− 1.5 (− 3.5;0.5)
CB	**− 3.8 (−5.7;-1.9)**	**−5.5 (−8.7;-2.2)**
Past asthma	−0.7 (− 2.0;0.6)	0.7 (− 1.5;2.9)
Current asthma	**−1.7 (− 2.8;-0.6)**	**−2.2 (−4.1;-0.3)**
COPD	**−5.6 (− 8.1;-3.1)**	**−4.6 (− 8.8;-0.5)**

^a^assessed through quantile regression models (estimating conditional medians of the response variable) considering PCS and MCS as dependent variables and respiratory diseases and characteristics of the sample as independent variables (adjusting for sex, age, BMI, educational level, smoking habits, non-respiratory comorbidities, cardiac comorbidities, study cohort and calendar period). Negative regression coefficients indicate a worsening in HRQL at an increase of the independent variable and vice versa

^b^*NAR* non-allergic rhinitis, *AR* allergic rhinitis, *CB* chronic bronchitis, *PA* past asthma, *CA* current asthma, *COPD* chronic obstructive pulmonary disease

All the values in bold are statistically significant (*p*<0.05)

The mental score was worse in CB (− 5.5, 95% Confidence Interval-95%CI: − 8.7; − 2.2), current asthmatics (− 2.2, 95%CI: -4.1; − 0.3) and COPD cases (− 4.6, 95%CI: -8.8; − 0.5) (Table 3), like in the case of PCS. Moreover, the AR and NAR mental score did not differ from the controls, similarly to the PCS.

## Discussion

In our study, respiratory disorders seemed to exert an influence on HRQL. Of importance, chronic diseases conditioned not only the physical, but also the mental health of affected subjects. This result is in line with other studies [1, 29], where scores of individuals with chronic conditions were lower (worse) than those of individuals not reporting any of the conditions studied.

Subjects suffering from COPD had the worse physical and mental health scores. This result seems to be of particular interest since it derives from a general population study. In their review, Joshi et al. [21], highlighted that COPD causes disabling physical conditions and psychological distress similar to those of cancer. Many patients with the most severe form of COPD suffer from depression, anxiety and panic, as a result of their physical impairment and social isolation [34, 35]. Recent studies have found that symptom burden of cancer and of severe COPD are similar [21, 36, 37].

In this study, subjects suffering from CA had a lower PCS and MCS than controls. In a European study of 864 asthmatic subjects conducted by Siroux et al. [38], asthma-severity was a predictor of PCS score of SF-36 quality of life questionnaire, but not of the MCS one. Ford et al. [39], in a US study conducted on 163,773 adults, found that people with CA have a worse HRQL than past asthmatics (ever asthmatics without “current” symptoms) and than subjects who have never suffered from it. Legorreta et al. [40] reported that, among 5580 patients with asthma aged 14 to 65 years, the mean scores for eight subscales of functional status were lower than those reported by the general population. Furthermore, the authors found significant decreases in functional status with increasing severity of asthma.

According to our hierarchical classification of respiratory diseases, a substantial percentage of subjects with CA and PA also suffered from AR and NAR. We found that rhinitis in subjects without asthma had no significant impact on HRQL in our study. In fact, controls had similar scores. This finding is not in line with other studies [7, 15] and ARIA guidelines [14]. This was probably due to the fact that asthma was also present in patients with allergic and non-allergic rhinitis in these studies.

The particularity of our survey was to study subjects with NAR and AR without past or current asthma and consequently, it was possible to assess the rhinitis effect on HRQL, without the interference of any respiratory comorbidity. Even if the physical and mental scores of the subjects suffering from rhinitis were lower than controls (except PCS for NAR cases), this difference was not statistically significant and it can be hypothesized that it is asthma that impairs HRQL, and not rhinitis alone.

Subjects suffering from CB had a lower physical and mental health than subjects without any respiratory disorder. It is of interest that the decrease in PCS and MCS is one of the highest among the considered respiratory diseases (Fig. 2). This is a significant finding since there are few studies [22] evaluating quality of life in subjects with cough and phlegm but without bronchial obstruction and no one compared quality of life scores among different respiratory diseases. Clinically, the presence of respiratory symptoms among subjects with preserved lung function may offer opportunity for interventions potentially improving quality of life. Our findings could also suggest that the exclusion of GOLD stage 0 from the 2006 update might be re-considered. The association found by Marcon et al. [26] of a reduced 6 min walking distance in subjects suffering from CB gives a potential and partial explanation of our findings.

In a Serbian study [11], the authors demonstrated that subjects suffering from CB and/or emphysema perceived their health status as bad/ very bad, with respect to controls. In another population-based survey conducted in Finland [12], the people with CB lived a worse daily life compared with the general population, and they had poor physical health.

It is not well established whether the differences in quality of life within subjects with respiratory diseases and controls are stable over time. In a study on the stability of normative data for the SF-36 in a sample of the general middle-aged Canadian population, mean SF-36 scores were found to change only slightly over three years [41]. It is possible that the variation could be greater in subjects with respiratory diseases, but to our knowledge, no longitudinal studies comparing directly healthy people and subjects with respiratory diseases have been done.

This study was performed on a sample from the general population of a single Italian centre, so the number of subjects with a particular disease was sometimes small: this fact could have somehow compromised the generalizability of the study.

To our knowledge, this is the first population-based study that investigates to what extent the most common chronic respiratory disorders affect HRQL. Unlike previous studies, we were able to consider several diseases at the same time by using a multicase-control design and a hierarchical classification of disease status. Furthermore, it is of note that this is the first study using SF-36 questionnaire in assessing simultaneously HRQL in a set of different respiratory diseases. We found that subjects who suffered from COPD, CA or CB had the poorest HRQL. On the whole, these findings emphasise that, even at the mild level of severity that is common in the general population, COPD and asthma have a significant impact on HRQL. Moreover, our data indicate that also CB, with lower impact on DALYs in comparison to COPD or CA [4] is not a trivial condition. It derives that clinicians should also carefully consider CB in relation to HRQL of these patients.

Moreover, our results highlights that not only physical but also psychological dimension of subjects with chronic respiratory diseases should be considered in clinical practice.

## Additional files

Additional file 1: Definition of cases and controls (a) and clinical tests (b). (DOCX 171 kb)

Additional file 2: Comorbidities among respiratory diseases. **Figure S1.** Comorbidities* between respiratory diseases and COPD cases. **Figure S2.** Comorbidities* between respiratory diseases and current asthma cases. **Figure S3.** Comorbidities* between respiratory diseases and past asthma cases. **Figure S4.** Comorbidities* between respiratory diseases and CB cases. (DOCX 117 kb)

## Abbreviations

AR: Allergic Rhinitis
CA: Current Asthma
CB: Chronic Bronchitis
COPD: Chronic Obstructive Pulmonary Disease
DALY: Disease Adjusted Life Years
ECRHS: European Community Respiratory Health Survey
FEV1: Forced Expiratory Volume in 1 s
FVC: Forced Vital Capacity
GEIRD: Gene Environment Interactions in respiratory Diseases
HRQL: Health Related Quality of Life
ISAYA: Italian Study on Asthma in Young Adults
MCS: Sf-36 Mental Component Score
NAR: Non Allergic Rhinitis
PA: Past Asthma
PCS: Sf-36 Physical Component Score
SF-36: Short Form-36 Health Survey
