# Supplementary material for: Health-related quality of life varies in different respiratory disorders: a multi-case control population based study
Source: BMC Pulm Med. 2019 Feb 7;19:32. doi: 10.1186/s12890-019-0796-8 (PMC6367788; doi:10.1186/s12890-019-0796-8)
Supplement: Supplementary file 1 — Definition of cases and controls (a) and clinical tests (b). (DOCX 171 kb) [file 12890_2019_796_MOESM1_ESM.docx]

**Appendix S1 - Definition of cases and controls (a) and clinical tests (b)**

**a) Definitions of probable cases of chronic bronchitis, asthma, rhinitis and probable controls based on the answers given by subjects to the screening questionnaire (available on** [**www.geird.org**](http://www.geird.org) **website).**

Probable cases of COPD/chronic bronchitis: he/she was at least 35 years old and reported plus at least one among chronic cough and phlegm (at least three months each year, for ten or more years), diagnosis of COPD, emphysema, chronic bronchitis, Hospital admission for respiratory disease in the last 10 years, Daily wheezing.

*Probable cases of asthma*: he/she reported a history of asthma and/or he/she reported asthma-like symptoms for asthma in the last 12 months and/or current use of asthma medicines;

*Probable cases of rhinitis*: he/she reported (for at least two years) any nasal allergies including hay fever and/or lifetime problem with sneezing, or a runny or a blocked nose during cold or flu; plus he/she had these symptoms currently or he/she took medicines for rhinitis; plus he/she had not sinusitis and polyps;

*Probable controls*: he/she did not suffered from wheezing or whistling, chest tightness, shortness of breath, asthma attacks in the last 2 months, lifetime asthma, nasal problems, cough and phlegm on most days for a minimum of 3 months a year and for at least 2 successive years, chronic bronchitis, chronic obstructive pulmonary disease (COPD) or emphysema diagnosed by a doctor, dyspnea and he/she didn’t visit an emergency room or he/she didn’t spend at least one night in hospital because of breathing problems in the last 10 years.

## Participation in GEIRD stage 1

In the Verona centre, the screening questionnaire was sent to 7,583 subjects,. Overall, 5,411(71%) subjects answered the questionnaire (Figure S1), and 5,233 of these subjects consented to participate in GEIRD stage 2. On the basis of their answers to the screening questionnaire, the subjects were hierarchically classified as follows:

1. subjects with symptoms suggestive of chronic bronchitis/COPD (n=282)
2. subjects with symptoms suggestive of asthma (n=682)
3. subjects with symptoms suggestive of rhinitis (n=725)
4. subjects without respiratory symptoms (n=2,062)

## Sampling for and participation in GEIRD stage 2

COPD, asthma and chronic bronchitis had top priority in the GEIRD study. Therefore, all the subjects with symptoms suggestive of chronic bronchitis/COPD and asthma were invited to the clinics. A 44% random sample of the subjects who reported symptoms of rhinitis, and a 61% random sample of the subjects without respiratory symptoms were selected for the clinical stage. The sampling fraction for the latter was chosen to balance asthma cases (which were the most numerous group of cases). The sampling fraction for the group of subjects with symptoms of rhinitis was the maximum possible sampling fraction after financial and logistical considerations^1^.

## b) Lung function and allergologic tests

Subjects underwent forced spirometry according to the American Thoracic Society reproducibility criteria^2^ FEV1 % predicted [Forced Expiratory Volume in the 1^st^ second] and the lower limit of normal (LLN) for the FEV1/FVC (Forced Vital Capacity) were calculated on the basis of Quanjer et al. equation^3^. Subjects with a FEV1/FVC ≥70% and ≥LLN underwent methacholine challenge test, if they consented. Persons with a FEV1/FVC <70% or <LLN underwent bronchodilator challenge test, and were invited (if eligible) to undergo methacholine challenge test on a second occasion.

Atopy was assessed by SPT using Bousquet et al. practice^4^.

Positive histamine and negative diluent controls were used. A reaction of 3 mm greater than the negative control was regarded as positive. A subject was considered to be atopic if he/she was positive to at least one out of 14 allergens (*Alternaria tenuis, Ambrosia artemisifolia, Artemisia vulgaris, Betula verrucosa, cat fur, Cladosporium herbarum, Corylus avellana, Cupressus arizonica, Dermatophagoides farinae, Dermatophagoides pteronyssinus, dog dandruff, Olea europea, Parietaria judaica, and Phleum pratense*) (ALK diagnostics, Denmark).

1. de Marco R, Accordini S, Antonicelli L, et al. *Int Arch Allergy Immunol*. 2010;**152**:255-63. ([www.geird.org](http://www.geird.org)).

2. Standardization of Spirometry, 1994 Update. American Thoracic Society. Am J Respir Crit Care Med. 1995 Sep;152(3):1107–36.

3. Quanjer P, Tammeling G, Cotes J, Pedersen O, Peslin R, Yernault J. Lung volumes and forced ventilatory flows. Report Working Party Standardization of Lung Function Tests, European Community for Steel and Coal. Official Statement of the European Respiratory Society. Eur Respir J Suppl. 1993;16:5–40.

4. Bousquet J, Middleton E, Reed CE, Ellis E, Adkinson NF YJ. In view methods for study of allergy. In: Allergy Principles and practice, Ed 2008 3rd Edition. 2008. p. 427

Figure S1: Subject selection from the screening stage to the clinical stage


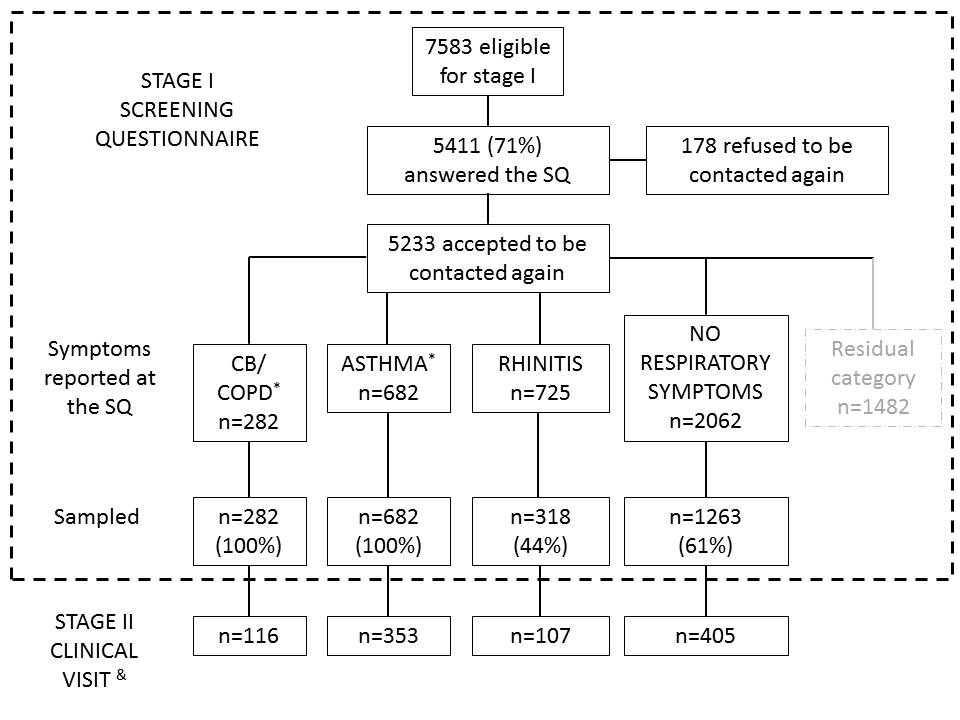


* At the screening stage, subjects were hierarchically classified, thus subjects in the group of chronic bronchitis (CB)/COPD could also have reported symptoms of rhinitis (n=74, 26%), and subjects in the group of asthma could also have reported symptoms of rhinitis (n=263, 39%) or CB/COPD (n=151, 22%).

^&^ With information on SF-36
