# Supplementary material for: Health-related quality of life varies in different respiratory disorders: a multi-case control population based study
Source: BMC Pulm Med. 2019 Feb 7;19:32. doi: 10.1186/s12890-019-0796-8 (PMC6367788; doi:10.1186/s12890-019-0796-8)
Supplement: Supplementary file 2 — Comorbidities among respiratory diseases. Figure S1. Comorbidities* between respiratory diseases and COPD cases. Figure S2. Comorbidities* between respiratory diseases and current asthma cases. Figure S3. Comorbidities* between respiratory diseases and past asthma cases. Figure S4. Comorbidities* between respiratory diseases and CB cases. (DOCX 117 kb) [file 12890_2019_796_MOESM2_ESM.docx]

**Appendix S2 - Comorbidities among respiratory diseases**

**Figure S1:** *Comorbidities* between respiratory diseases and COPD cases*

* COPD=Chronic obstructive pulmonary disease; CA=current asthma; PA=past asthma; AR=allergic rhinitis; NAR=non-allergic rhinitis; No resp. com.=no respiratory comorbidities.

**Figure S2:** *Comorbidities* between respiratory diseases and current asthma cases*

* COPD=Chronic obstructive pulmonary disease; CA=current asthma; AR=allergic rhinitis; NAR=non-allergic rhinitis; No resp. com.=no respiratory comorbidities.

* * Cases into dash boxes are considered as COPD cases (n=16), since subjects were hierarchically classified and phenotyped.

**Figure S3:** *Comorbidities* between respiratory diseases and past asthma cases*

* COPD=Chronic obstructive pulmonary disease; PA=past asthma; AR=allergic rhinitis; NAR=non-allergic rhinitis; No resp. com.=no respiratory comorbidities.

* * Cases into dash boxes are considered as COPD cases (n=5), since subjects were hierarchically classified and phenotyped.

**Figure S4:** *Comorbidities* between respiratory diseases and CB cases*

* CB=chronic bronchitis; AR=allergic rhinitis; NAR=non-allergic rhinitis; No resp. com.=no respiratory comorbidities.
